# *“We Tried to Take Care of Her, but it Got Too Exhausting”*: A Study of the Transition From Family Carer to Employer

**DOI:** 10.1177/23333936231202876

**Published:** 2023-10-16

**Authors:** Tove Mentsen Ness, Wasiq Silan

**Affiliations:** 1 Nord University, Levanger, Norway; 2 University of Helsinki, Finland

**Keywords:** Indigenous peoples, Tayal, family carer, employer, live-in carer, vulnerability, care work, Taiwan, 原住民族, 泰雅族, 家庭照顧者, 雇主, 外籍家庭看護工, 脆弱性, 長期照顧, 台灣

## Abstract

In Taiwan an increasing number of families are employing live-in carers from abroad to cope with care responsibilities, including the Indigenous Tayal. The aim of this research was to understand the transition from Indigenous family carer to employer with older family members who have extensive care needs. Six Indigenous employers were interviewed, and a narrative hermeneutic analysis was performed. The Tayal caregivers’ cases revealed that their transition to employing live-in carers was complex and filled with ethical dilemmas due to their vulnerable positions. They tried to ensure person-centered care for their family members, but by doing this they risked reproducing vulnerability when transferring their own vulnerability to the live-in carer. The results indicate the interwoven nature of care dependency when it is defined by multiple vulnerabilities, Indigeneity and migration, and the multifaceted components of cultural safety.

## 摘要

在台灣，越來越多的家庭聘請外籍家庭看護工來分擔照顧責任，其中包括原住民泰雅族的家庭。 本文旨在了解這群照料重度需求長者的原住民家庭照顧者如何過渡成雇主的角色轉換歷程。我們採訪了六名原住民雇主，並進行了敘事解釋學分析。 結果表明，家庭照顧者聘請外籍家庭看護工的角色轉換是複雜的，並且由於其脆弱境地而充滿道德困境。 家屬想用以人為本模式來照顧長者，所以聘請外籍家庭看護工。但，在聘僱時卻將自己的脆弱性轉移給她們，造成脆弱性被複製的風險。本文結論揭示了照顧依賴的交織特性，以及細究多重脆弱性、原民性與遷徙，還有文化安全的多元概念與內涵如何形塑照顧。

## Introduction

Indigenous peoples have suffered disproportionate discrimination, oppression, marginalization and exploitation throughout the world (Cobo, 1983), including in Asia (Tauli-Corpuz, 2020). Based on this shared experience, the conceptual understanding of legal meaning of the term Indigenous peoples is constructed (Scheinin, 2004). Meanwhile, the marginalization has harmed Indigenous peoples, for example in terms of health international studies show that Indigenous communities have overall poorer health and social outcomes than the benchmark population (Anderson et al., 2016; Bartlett et al., 2007). Moreover, the standardized healthcare services they receive are of lower quality (Browne & Fiske, 2001; Tang & Browne, 2008) and culturally unsafe (Blix & Munkejord, 2022; Ramsden, 2002). Hokanson et al. (2018) furthermore stress that Indigenous care workers have poorer health and a higher care burden than non-Indigenous caregivers. We also know that providing care to older family members with extensive care needs is demanding, see, for example, Y.-N. Wang et al. (2018). Doing so without formal support is even more demanding, and often results in increasing stress, fatigue, burden and ultimately vulnerability for the family carer (Munkejord et al., 2020; Proot et al., 2003). These factors have compounded the care burden on families with Indigenous backgrounds.

The Tayal, the central case in this article, are one of the officially recognized larger Indigenous peoples in Taiwan, consisting of approximately 93,000 persons (Council of Indigenous Peoples, 2022, https://www.cip.gov.tw/zh-tw/news/data-list/940F9579765AC6A0/index.html?cumid=940F9579765AC6A0). The Tayal people have had their own distinctive cultures, languages, customs and social structures, but the colonial powers –in particular the Chinese and the Japanese—imposed military, economic and cultural colonization that threatened their community survival (Gao, 2021). In 2016, Taiwan’s president Tsai Ing-wen officially apologized to the Indigenous peoples, but the ongoing colonial legacies that threaten Indigenous communities are still visible in care for older persons (F. T. Y. Wang & Yang, 2017) and land occupation (Kuan & Lin, 2008). Christianity has substituted the traditional Gaga (Tayal law) and Utux (omnipresent spirits) system that was the Tayal’s dominant morality and way of life. To date, it is commonly believed that over 80% of the Tayal are Christian, although the version of Christianity in question may vary depending on how much the local church integrates Tayal cultural elements (cf. Ru & Lo, 2015). To better contextualize the Tayal case, we need to understand the long-term care system in Taiwan so as to see the particular set of care burden faced by Tayal family carers.

### The Context of Taiwan’s Care System for Older People

In Taiwan, as in many countries in Asia, family care is considered an ideal (Liang, 2011), while sending older people off to nursing homes remains less desirable (Chou et al., 2015). Public-funded home-based care services hardly exist (Chou et al., 2015), which means that Indigenous communities often end up caring for their older family members at home by themselves. Filial piety, a cultural ideal, obligates families in Taiwan to take on caring roles (Cheung et al., 2020), and is often shown in Indigenous families. It is a traditional belief that parents sacrifice themselves for the children, and children repay when their parents age (Chung, 2001). This means that families represent a place for love, care and education for the children, and they repay with care for their parents and older relatives. The family therefore, from an idealistic perspective, becomes the an effective welfare system providing care and ensuring that basic needs and rights are respected and realized (Park, 2021). The responsibility for caring roles is written in Taiwan’s Civic Code, and it not only obligates children to care for their parents, but mandates those lineal relatives by blood, where husband and wife, siblings and the head and members of a household are all under a *mutual obligation* to maintain one another (F. T. Y. Wang et al., 2006). As filial piety is a codified responsibility, public-funded service only intervenes when families are in crisis and end up in vulnerable positions (Lan, 2002; Liang, 2018). In recent years, this filial model has become less and less sustainable due to the rapid transformation of Taiwan’s family structure (F. T. Y. Wang et al., 2006), as one-person and nuclear households now comprise nearly 80% of the population (Directorate-General of Budget; Accounting and Statistics, 2021). Several ongoing long-term care reforms, such as the Ten-year Long-Term Care Plan 2.0, have been implemented as a response to support these families. This long-term care plan is intended to support families by increasing the number of care service items (from eight to 17 items) and by making public care more assessable to individuals experiencing vulnerability (care users are estimated to increase from 511,000 to 738,000 persons). A government-commissioned survey claims that over 90% of the family carers are satisfied with the current services they received (Ministry of Health and Welfare, 2021), but this may not be the case for the indigenous communities (Hong, 2022). Family carers are trapped in the transition between the filial model and a publicly-funded care model. The lack of formal care services and the change to family structures have given rise to the phenomenon that many family caregivers who are already old themselves are caring for even older members of the family at home without proper support. In fact, 37% of family carers are 65 years and above, with over half of them caring for their family members more than 10 hr a day for an average of about 8 years (United Daily News, 2022). There is an emerging evidence base indicating that an overwhelming filial burden can place family carers at risk of committing domestic violence, suicidal experiences or even homicidal thoughts and behaviors (Jeang et al., 2016; Li, 2020; O’Dwyer et al., 2021).

In Taiwan, family carers employ migrant workers to reduce the overwhelming stress and demands of caring for older family members (Basnyat & Chang, 2017; Munkejord, Ness, & Silan, 2021). In fact, 30% of Taiwanese families who have a family member with extensive care needs have chosen to employ live-in carers (Taiwan Association of Family Caregivers, 2021). Since 1992, migrant workers have been permitted to enter Taiwan to work, among other roles, as live-in carers (Liang, 2018), and this has increased dramatically over the years. To date, 76% of migrant care workers, some 170,000 persons, have come from Indonesia, in addition to some 28,000 persons from Vietnam and 26,000 from the Philippines (National Developmenet Council, 2021). Only 6% of them work in long-term care institutions, which entitles them to labor law protection; more than 90%, however, are employed as 24-hr live-in carers in private households without any legal protections (Ministry of Labor, 2022). The wages of these carers have been low, and their working conditions are often difficult due to long hours and no regular days off (Liang, 2018). In fact, human rights violations perpetrated against migrant workers is one of the principal human rights issues in Taiwan due to the exploitative brokerage system (United States Department of State, 2022, https://www.state.gov/reports/2022-country-reports-on-human-rights-practices/taiwan/; Huynh & Cheng, 2022) and the absence of equal protection (C.-F. Chen, 2016). The private brokers not only charge migrant workers hefty fees for matching them with Taiwanese employers, but also demand a recurring monthly payment for services that many say do not exist (Huynh & Cheng, 2022). Although the system is highly exploitative, migrant live-in carers have nevertheless become indispensable to Taiwan’s care system in supporting older adults (Liang, 2017; Munkejord, Ness, & Silan, 2021).

Unable to cope with the increasingly extensive care needs, family carers often turn to the employment of a live-in carer in the absence of a government formal service (Chien, 2018; Munkejord, Ness, & Silan, 2021). In Taiwan, the majority of people with extensive needs are cared for by their family and migrant live-in carers (Chou et al., 2015). Although hiring live-in carers is prevalent among wealthier Taiwanese families, an increasing number of less affluent families are also opting to hire migrant live-in carers, and this includes Indigenous families.

Challenges related to family care among Indigenous Peoples in Taiwan are striking yet have not been elucidated. Indigenous Peoples refers to those Austronesian speakers who have been living in Taiwan for thousands of years. The military, economic, cultural and spiritual colonization imposed by settler colonizers in the past 400 years has had a severe and profound impact on the wellbeing of Indigenous Peoples today (Wang, 2011). Indigenous populations are growing older: 8% of the Indigenous population in Taiwan is over 65 years of age (National Health Research Insitutues, 2022, pp. 7,12-13) signaling that an increasing number of Indigenous families are coping with elders with extensive needs. Families living in Indigenous communities, like those in the rest of Taiwan, end up arranging care and support for their family members since, as mentioned above, caring for older people with extensive care needs is considered a private issue (Huang et al., 2012; Liang, 2014; F. Wang, 2010). In addition, Indigenous families utilize less formal long-term care services because formal services are perceived culturally unsafe (Ru, 2018; F. T. Y. Wang & Yang, 2017); too complicated and fragmented to apply; inconvenient to access (Kasirisir, 2015); and too expensive to use (F. T. Y. Wang & Yang, 2017). Indigenous families therefore end up keeping their older family members at home as long as possible without the help of public community- and home-based care. Hence, families—both Indigenous and non-Indigenous—tend to rely on migrant live-in carers to enable them to keep older family members living at home and in the community.

With few exceptions (Basnyat & Chang, 2017; Chiatti et al., 2013; Degiuli, 2010; Hoens & Smetcoren, 2023; Salami et al., 2017), the experiences of families who hire migrant live-in carers have not been studied. Although Lan (2004) and Liang (2021) have aptly contributed to the literature concerning the experiences of Taiwanese families, the key insights relating to Indigenous families who employ live-in carers remain unaddressed. In fact, migrant workers are usually framed as *competing* for work with Indigenous Peoples rather than being employed by them (Liuhuang & Hsin, 2018). This is because Indigenous Peoples are usually categorized as a vulnerable group with multiple disadvantages (e.g., poverty, low education, low skills) (Liuhuang & Hsin, 2018; Ru & Wang, 2016). Further examination of Indigenous family carers’ experiences is therefore urgently needed for three reasons: First, to address the gap in the literature through enhancing our understanding of the transition from family carer to employer among Indigenous families in Taiwan with older family members that have extensive care needs; second, to provide a more complete picture that helps explain the interrelationships and collaborative efforts between live-in carers and their employers, as previously examined by Munkejord, Ness, and Silan (2021); and third, on a more distal level, to further examine the links to the bigger question concerning how intergenerational trauma, social inequities and the ongoing settler colonial policy structure impact Indigenous peoples’ care and wellbeing (Teyra & Hsieh, 2022; F. T. Y. Wang & Yang, 2017). Therefore, we ask: What does it mean to employ a migrant live-in carer in an Indigenous family? How does the process of employment play out when two groups suffering from vulnerability are involved, namely, the migrant live-in carer and the Indigenous families? What are the implications?

## Method

### Design

In this project, we aim to gain a better understanding of the transition from family carer to employer among Indigenous families in Taiwan with older family members that have extensive care needs through a qualitative interpretative and constructivist research design (Charmaz, 2006; Haavind, 1999). This approach allows us to delve into the families’ sense-making, as the reality is seen as co-created when we encounter each other. The Tayal people were the focus of the study, which is part of a larger project financed by the Research Council of Norway that brings together the issues of aging and care in the Indigenous communities in Tayal territory and the Sápmi. One of the members of the research team had grown up in a Tayal community in Taiwan; therefore, selecting the Tayal as the focal point of the study provides us in-depth insider knowledge and access to relevant participants. We see including an Indigenous Tayal researcher on the team indispensable for co-creating a form of decolonial knowledge centering aging and care (Chaouni et al., 2021). In line with Indigenous methodologies that underline ethical engagement with Indigenous communities, the research team invokes relational accountability (Wilson, 2008) to ensure trustworthiness in the research process.

### Sampling and Participant Selection

During a field trip in the fall of 2019, we interviewed 11 employers and 10 migrant live-in carers providing care to older persons with extensive care needs in a Tayal community in Taiwan. In most cases, this study also includes our reflection based on our interactions with the older person receiving care. This article is part of a larger research project (Munkejord, Ness, & Gao, 2021; Munkejord, Ness, & Silan, 2021). To examine the transition from family carer to employer among Indigenous families in Taiwan with older family members that have extensive care needs we focused on the six interviews with employers with a Tayal background that were collected in the larger study. One of those employers was the spouse of the care recipient, whereas three were a child of the care recipient, one was an in-law and one was the grandchild. The recruitment was done through convenience sampling (Etikan et al., 2016) in three different ways. First, via Wasiq Silan, the research team was invited to visit a Tayal families who had lived in the community for many generations. Second, the research team visited the local Day Clubs for older people in the area. As the Day Clubs offered activity to promote healthy aging, and functioned as community-based care centers, we were able to recruit a number of older persons. Third, the parents of Wasiq Silan, who had lived in the community for decades and were active in the local church, introduced the research team to potential participants in the community. These three ways led to information about the project being distributed in the Tayal community where participants were recruited. All participants in this study are given pseudonyms.

### Ethical Considerations and Roles in the Research Team

Before taking part in this study, oral consent was obtained from the participants, and they were also guaranteed anonymity. In order to protect their identity, no real names were transcribed or asked for during the interviews. Instead, general titles and identification numbers such as employer one, employer two, etc., were used. This study was assessed by Norwegian Agency for Shared Services in Education and Research (project number 577949) and carried out in agreement with the Declaration of Helsinki (World Medical Association, 2013). Both authors contributed equally to the analysis of the data and the writing and revision of this article and are therefore named in alphabetic order.

### Interviews

The interviews gathered stories that were embedded through substantial and meaningful narratives (Riessman, 2008). Participants were asked questions concerning why they as a family decided to hire a live-in carer, when they made the decision and how the process evolved. Additionally, they were asked about their experiences of having a live-in carer in their family. These questions then provided us with the individuals’ challenges regarding their transition from carer to employer. The interviews were planned, performed and transcribed by Mai C. Munkejord in collaboration with the authors of this article, who were both present during the interviews. Author Tove M. Ness listened and asked some questions at the end of each interview, and author Wasiq Silan, in addition, acted as a translator (English–Mandarin Chinese). The interviews were performed in Mandarin Chinese because the participants in this study did not speak English. Because author Wasiq Silan was the only one that could understand Mandarin Chinese, she acted as a translator for both the participants and the other researchers. The interviews were recorded and transcribed and lasted from 25 min to 2 hr, with an average of approximately 1 hr. Most interviews were conducted in the participants’ home, the care recipients’ home or another place that the participants preferred, for example, in a café.

### Data Analysis

The data analysis draws from narrative hermeneutic analysis (Ricoeur, 1976; Wiklund et al., 2002) and three levels of reading (Lindseth & Norberg, 2004). In the following, we will explain how the analysis was performed to allow readers to assess the trustworthiness and relevance of the findings (Stige et al., 2009). In the initial round of analysis, we enhanced our analytic and interpretive awareness by conducting several readings and reflecting on them in order to become familiar with the data. We attempted to grasp the meaning of the data, utilizing not only transcripts, but also field notes. We wrote and used the fieldnotes in order to more fully understand the context of the participants in this study. In addition, a way of remembering where we were and how the field was experienced by us as researchers. Second, we met both physically and remotely several times to identify the narrative structures, first by focusing on how the participants structure their stories and thereafter by exploring the horizons of their embodied experience (Frank, 2013), or their lives as lived experiences before their formulation into spoken narratives (Russo, 2021). In the last step of the analysis, we read the data again in their entirety during the first stage and examined the structures of the plots during the second. With a reflexive and open mind, we critically examined how our preunderstanding, research values, experience and training impacted the process. The authors arrived at possible narratives of transition by carefully reviewing the reasons for the transition and existential experiences like hope, fear, suffering, and moral dilemma in order to explore why they chose to become employers. The hermeneutic process over time, as well as between parts and whole, observer and the viewer, enables a deeper and more nuanced understanding of the families’ transition to employing a live-in carer. We are cognizant of the fact that there are many ways to interpret the participants’ transitions, as Lindseth and Norberg (2004) stress that a text has not only one meaning, but are multifaced and shaped by the readers possible perception and awareness of alternative possibilities.

Eventually, three stories of particular clarity and distinctness were identified to amplify the difficult transition from carer to employer: (1) Kawas, from exhausted carer to demanding employer; (2) Maya, from pained carer to empathic employer; and (3) Yuma, from strained carer to faithful employer. The three types of transitions signify three distinctive Indigenous families and their varying transitions into the live-in carer system. The three stories represent the transition of three of the participants in the study, and can therefore be seen as case studies (Yin, 2014). Further, these three cases capture key elements of the complex dilemmas that were evident in the transitions experienced by the other three Indigenous interviewees.

## Result

The Indigenous older persons in this study were cared for by foreign live-in-carers who had little or no knowledge about their cultural knowledge, did not speak their language, and did not know how to facilitate healthcare services when they came to the families; rather, they had to learn while working in the families. None of them had any professional background in healthcare. In the following, we present examples of narratives that capture three types of transition from family carer to employer identified in the data.

### Kawas: From Exhausted Carer to Demanding Employer

Kawas was in her mid-sixties, and she had to take care of her parents who lived nearby together with her three siblings. Her mother and father were both in their 80s, and they have lived all their lives in the Tayal territory. The situation started with the mother in the family experiencing a stroke, which led to a hospital admittance and thereafter the need for extensive 24/7 care. Even though her children experienced their mother as confused, restless and easily angered, Kawas explained that they tried to tolerate her behavior and share the care tasks between them. They rotated every week and took shifts to provide care. Kawas, who lived nearby with her husband, children and grandchildren, explained:

[W]e were full of love in the beginning, but then it was difficult to care for [my mother] because she was always angry, so it was very complicated. We took turns, one months, two months, three months like that. Towards the end we were really tired and we started to point fingers at each other, like: Why do I have to take so much responsibility? Where were you? So we used to be one big family, but then we started to point fingers at each other.

The community had a Day Club for elders held by a local church and funded by the Council of Indigenous Peoples. It was open and free of charge to everyone, running activities 5 days a week in the mornings. However, Kawas expressed concern that the Day Club was too temporary and fragmented to respond to their mother’s needs. In search of a sense of continuity of care, they saw no other choice but to hire a live-in carer. This was not that easy, as Kawas’s father, coming from an Indigenous Tayal chief’s family, told his children *“As Tayal we are supposed to take care of each other in this family, and we do not need anybody from the outside.”*

This created a dilemma for this participant—a choice that was made particularly difficult because she was unable to fulfill the expectations of this cultural ideal. In addition, it is to be noted that hiring a live-in carer becomes viable because many in this family had access to a pension, which was not the case for the majority of the Indigenous peoples living in the village.

In short, the participant in this transition faced layers of dilemmas that included both emotional and cultural elements when transitioning away from a family carer. The emotional dilemma, which is closely interlinked with cultural expectation, remained strong throughout the whole process, as she felt they were obligated, and desired to, care for their mother by themselves, but were physically and mentally unable to do so. Hiring a live-in-carer seemed to be the only option, as Kawas explained:

Before the first live-in carer we, my brothers and my sisters-in-law and I, everybody took turns, like helping my mother to bed, sleeping with her, we took turns. But we could hardly sleep, because my mother would scream, so it was difficult. It was like insomnia. . . so after 3 months, we got a live-in carer.

Another intertwined dilemma pertained to trust and control. The participant expected the live-in carer to provide excellent care, and expressed frustration in seeing the live-in carer use her headphones all the time and could not hear their mother when she needed help. Kawas exemplified this by saying:

One time my mother was not taken to the toilet in time, so she peed in the bed. That is a sign that [the live-in carer] does not really live up to the expectations. . .. So I will continue to go there all the time to check up.

Here, Kawas acknowledged the ambiguity of her new role as an employer. Worried that her mother was not treated well, she had therefore considered strengthening control over the live-in carer. Kawas explained:

In the beginning, she did not take good care of my mother, because she did not hear when my mother called out her name. . .. I have considered putting a camera in the house.

To summarize, the family carer faced an emotional and cultural dilemma when she and her siblings tried to cope by themselves and live up to the standard of the ideal family, but this was ultimately unsuccessful because of her mother’s extensive needs. Becoming an employer brings dilemmas concerning communication as well as trust and control, as the connection between the live-in carer and her mother was perceived as lacking. The challenges were temporarily settled when Kawas decided with her siblings to find a new live-in carer whom they hoped would be more willing to establish connection with their mother.

### Maya: From Pained Carer to Empathetic Employer

This transition included a care recipient with dementia and her children. Maya, while working full time as a hairdresser, took care of her mother while her brother, a bus driver who occasionally hunts, shared the caring load after his divorce. The tipping point emerged as Maya started to notice that her mother was behaving in a manner that she did not understand and sensed things that were not functioning properly. Maya’s mother was later diagnosed with dementia. Maya was alone to care for her mother for some years because her siblings lived elsewhere. One or 2 years after her mother was diagnosed with dementia, the situation quickly worsened and Maya realized her mother needed 24/7 care. As she had to work, she asked her aunt to look after her mother, but this did not go as planned because her mother was suffering from severe memory loss and her aunt did not realize her mother was ill. This created an emotional dilemma, as Maya explained:

I asked my aunt, my mother’s sister to take care of her. In the beginning it was fine, but the second month was really painful; they were insulting each other, and the worst thing is that my aunt thought that my mother was normal, so she tried to reason with her, it was really frustrating.

Because Maya’s aunt did not understand her mother’s situation, Maya turned to the long-term care services in the community for help. Unfortunately, Maya experienced the public elderly care services as severely inadequate and “*lacking empathy*” and resulted in an emotional dilemma in relation to ensuring her mother’s care. Maya explained:

One day my mother went there, I got a phone call from this government service saying that “your mom has a problem, and we cannot really deal with it,” and I feel that they lacked empathy, I feel that you really need love. I feel that you need to have experience, so that when they take care of old people, then we, as family, can really feel that the old people are really taken good care of. They cannot really treat it as work only. I was angry to death about that.

The formal care system failed Maya, and she felt left alone in carrying out the care responsibilities. Another interrelated aspect of the emotional dilemma that Maya experienced was the way her mother’s sleep disturbance was treated. The physician recommended that the care recipient take sleeping pills for her sleeping problems, but without much success. The sleeping pills had negative side effects that bothered Maya: “*After [my mother] woke up, she would stare into nothingness, and it hurt us, because we wanted her to sleep, but we did not want her to be like that.”*

Caring alone for her mother with dementia was extremely demanding, both physically and emotionally, thus the only solution was to hire a live-in carer. This decision involved Maya and her brother, as their other siblings did not participate in the care. When asked about the role of her brother in the caring routines, the participant pointed out that, although the brother contributed financially and shared his car, his level of engagement was influenced by his participation in Indigenous traditional practices, such as hunting. Maya said:

Yesterday, it was supposed to be him that was going to take my mother to the hospital to see the psychiatrist, but it turned out to be me because he was not yet home from hunting.

The participant’s brother was enthusiastic about hunting and thus missed regular hospital visits. The lack of reliability from the brother presented a form of cultural dilemma. The transition to becoming an employer was difficult, as ideally she wanted the family to care for their mother on their own. As care needs became increasingly demanding, hiring a live-in carer turned out to be the only way to secure good care for her mother. She reflected on what it meant for her to become an employer:

It gives me a lot of comfort. So I don’t need to worry about what is happening at home. . ..I think that the most important thing is that [live-in carer] takes good care of my mother, and then she will be in my family.

Another dilemma that was prominent for the participant was the financial one, as she struggled to make ends meet in order to pay the live-in carer. Three of her five siblings contributed somewhat to the live-in-carer’s salary, but she not only shouldered most of the financial responsibility but also lived together with her mother.

Maya had employed several live-in carers and had faced a trust dilemma regarding some of them. As the live-in carers are assigned by the broker system, she could only hope for the best. It turned out that the current live-in carer was finally a good match. This live-in carer was from Indonesia and had a good relationship with her mother, as well as the ability to recognize her mother’s needs. This live-in-carer even performed every day domestic tasks, although Maya clearly highlighted that, despite this assistance, she expected the live-in carer to prioritize taking good care of her mother. When prompted about what routine the live-in carer had, the participant pointed out that the priority is to ensure that the live-in carer gets enough sleep. She approached her role as employer from the viewpoint that she should support the live-in carer since the carer is substituting for her, explaining: “*because it is very demanding to take care of my mom, I know it myself.”* It was evident that instead of behaving like a supervisor in a superior position, Maya took on a role as a team member who showed solidarity with the live-in carer. She also explained that she totally trusted the current live-in-carer:

My mom is afraid of cold, so I tell her that you can dip her feet into hot water. But other than that, I have not given any particular instructions. I mean, the important [thing] is to take good care of her.

As mentioned above, the participant highlighted that her main concern is the best care possible for her mother. In the beginning, she carefully observed how the live-in carer interacted with her mother, and over time learned she could trust this person. In fact, trust in the closely-knit Indigenous community is manifested in the fact that Maya’s mother even gave the live-in carer her own mother’s name as a sign that she was truly accepted in the family:

I also told my mom, “Yes, you can give her an Indigenous name.” And my mother said, “I want to give her my mom’s name, because my mother is also very beautiful, very gentle and very hard working.”

Even though the live-in carer gained the trust of her employer and was perceived as a family member, the fact that she was only temporarily in Taiwan to do care work worried Maya. As she explained: “*I see her as my family, she is like my sister and my friend. . . but what happens if she leaves? I don’t know.”*

In sum, the participant experienced dilemmas in the transition from family carer to employer. After struggling to manage her mother’s increasing care needs, made more challenging because they lived in the same home, the participant made the difficult decision to hire a live-in-carer.

### Yuma: From Strained Carers to Faithful Employers

Yuma was in her early sixties when she had to start taking care of her sister-in-law, who returned to the Indigenous village after 40 years of living abroad. Yuma recalled clearly the day when she received an urgent phone call from her sister in-law who had had a stroke. The stroke was the initiation of the crisis in this transition. Yuma’s sister-in-law had one daughter who lived abroad with her own family; therefore, she could not participate in the care of her mother in Taiwan. Hence, the caring responsibility and the cultural ideal of supporting one another fell on the family members who were living in the village. Yuma’s husband was ambivalent about taking care of his sister whom he thought should be able to independently organize her own care after living abroad for many years. Ultimately, Yuma took up the responsibility for initiating and organizing care for her sister-in-law.

Initially, Yuma and the rest of the family tried to cope with her extensive care needs on their own, but it became too exhausting. This created an emotional dilemma for Yuma, because she did not have the ability to take care of the family member, although she wanted to. Before they sent her to the nursing home, the family divided into groups and took shifts to care for her for 2 months, including feeding her with a tube, coping with her sleep disturbance, and helping her to rehabilitate. Yuma explained:

Because all of us had to work [it became too difficult]. We would have to change the diaper, and everything was heavy because she could not move her right leg, so she could not move anymore [despite the rehabilitation]. So we sent her to the nursing home.

Yuma and her family visited every other week after admitting her sister-in-law to the nursing home, because she thought it was important to be present even though they were physically separated. However, Yuma found out that her sister-in-law became unhappy without company at the nursing home, so Yuma and the family brought her back home after few months. Yuma explained the dilemma she faced in making this decision. She saw that the care recipient became increasingly lonely because of the absence of her own family. She noted that it was a kind of *loneliness from a family perspective*:

I feel that sometimes in my sister in law’s heart there was something missing. She would be wondering like “Where is my family?” She would of course not say it, but we can tell it from the from the way she looks at us.

The dilemma about her sister-in-law’s care had an emotional component, while at the same time reflecting a sense of moral aspiration. Yuma was uncomfortable with the fact that her sister-in-law was deprived of the warmth of family. In addition, she was also concerned about the quality of care and pointed out that nursing home staff cared for too many people and were too strict with their daily routine, concluding that they cared less about the people living there. The way to resolve this emotional dilemma was to hire a live-in carer, where Yuma could keep her sister-in-law close to the family and guarantee that someone looked after her and respond to her needs around the clock.

The participant highlighted that as a family they wanted the best care possible for their family member, so the decision to employ a live-in carer was straightforward. Yuma highlighted that she trusted the live-in carer and that she was perceived as a member of the family. Although the employer admitted that the live-in carer did a certain amount of household chores, a clear message was that she did not expect the live-in carer to take over her family’s responsibilities in addition to caring for her sister-in-law. Yuma acknowledged that trust may be an issue for other families employing live-in carers, but she did not see it as an issue for her family. Yuma noted:

The most important thing is to take care of my sister[-in-law]. Of course, a lot of live-in carers are in families where they put up monitors or cameras and they are afraid that the live-in carers come only for money, and without any love.. . . [I told the live-in carer] I trust that you will take good care of my sister, and you treat her as your own mother. . .. We treat the live-in-carer as part of the family.

Yuma further explained that in being an employer, one is faced with an option to trust or not trust the live-in carer. She is convinced that treating the live-in carer as part of the family is key. When asked about what makes a good employer, she answered: “*We are Indigenous people, and we don’t give the live-in carers too much work. So the majority of Indigenous people take good care of live-in carers.”*

Moreover, instead of encountering a cultural dilemma, the participant drew on her Tayal values and traditions to resolve the possible ethical dilemma that she might encounter as an employer. For example, the agency told the live-in carer that they are not allowed to sit at the same table and have a meal with their employers, but the participant pointed out that this was similar to the way slaves were treated in the U.S., and they strongly disagreed with this directive. Accordingly, her husband would invite the live-in carer, who would otherwise sit in the corner to eat by herself, to come eat with them. The participant Yuma explained:

We Indigenous peoples, we treat live-in carers as our family. Because after all, they come all the way here to earn money, to provide for their children, so we need to treat them well, we cannot treat them as maids.

In particular, the way of being a Tayal is closely interlinked with the core values of being a Christian. The family of the participant were active church members who attended Tayal Presbyterian church activities several times a week. The participant understands her role as an employer as embedded in the identity of being a Tayal and a Christian, which is about treating the live-in carer on an equal footing, that is, as part of the family. Therefore, when prompted about the question concerning resolving religious differences between the participant’s family and the live-in carer, she explained:

I don’t stop her practicing her religion. . .. we pray because we are Christians, and we pray for her, and when we come to pray [for my sister-in-law], we don’t force her to pray with us, but sometimes she comes to pray with us.

Yuma shared that transitioning from family carers to employers among Indigenous families with extensive care needs is challenging and involves multiple layers of dilemmas.

## Discussion

The aim of this research was to understand the transition from family carer to employer among Indigenous families in Taiwan with older family members that have extensive care needs. We additionally wanted to explore the experiences that influenced decisions to employ a live-in carer, and experiences of having a live-in-carer in the family. The narratives demonstrate that the transition from family carer to employer can be a distressing and complex process, and the participants experienced multiple ethical dilemmas relating to emotions, culture, finance, trust and communications in their transitions from family carer to employer. Additionally, the results indicate that when Indigenous family carers became employers, colonial relationships were reproduced in various forms of vulnerability. The three typologies of family carer experience show how structural factors and context shape seemingly similar reproductions of vulnerability that are played out in varying ways. All Indigenous family carers were women, of Christian faith, and they all lived in Indigenous communities, earning a small salary and participated in the exploitative migrant worker system to which the live-in carer belonged. They were dissimilar on the account of their family structure, the level of resources they could mobilize and their moral expectations for caring. The reproduction of vulnerability onto the live-in carer was most prevalent in the transition of Kawas, where she considered installing a security camera and felt that she should take a more demanding role in monitoring and managing the live-in carer. In Maya’s transition, the vulnerability exemplified is gendered, as Maya was a divorced daughter who returned home with a full-time job, and attempted to balance the exploitative migrant worker system with trying to alleviate the negative consequences of this system as much as she could. Lastly, Yuma’s transition shows how the Christian faith, a form of spiritual colonization across many Indigenous communities, was deployed in favor of the dignified treatment of both the grandmother and the live-in carer. All three transitions illustrate complex pathways colonial relationships are reproduced through forms of vulnerability. We explain more below.

### Indigeneity and Migration in a Vulnerability Quandary

Some populations may be seen as more vulnerable because they may need more humanitarian assistance or are excluded from financial and social services (Marin-Ferrer et al., 2017). However, it is to be noted that vulnerability is not fixed within a certain population, but we see it as a more of a dynamic phenomenon where anyone can be in a vulnerable situation, although some can be more entrenched in one than others (Kuran et al., 2020). In this study, one cannot deny that live-in carers are in an extremely vulnerable situation, as they are excluded from legal protection (Labour Standards Act), deprived of the freedom to change employers and obligated to work around the clock without proper rest (Ministry of Labor, 2022). However, family carers can also be in vulnerable situations as sufficient formal services are not available. They may be older people themselves, tied by a cultural code and obligated to carry the heavy burden of caring for their family members with extensive care needs, without rest, for many years. In fact, the moral condition of a family that is responsible for caring for an older family member—either by themselves or by employing others—entails a vulnerable situation for the participants. This means that family carers may reproduce vulnerability in the live-in carer when hiring them. This reproduction of vulnerability between the employer and the live-in carer is layered, complex and embedded in a mutual dependency that is interwoven, as previously elaborated by Munkejord, Ness, and Silan (2021). We should add that the interdependence is not only between the employers and live-in carers, but also the older family members with extensive care needs: they are being cared for by exhausted family carers who are juggling the responsibility of caring and full-time work. When the live-in carers are hired, the older family members are cared for by persons from foreign countries who have little or no knowledge of their cultural background, seldom speak the same language as the care recipients, do not know how to facilitate healthcare services to Indigenous care recipients and seldom have any professional background in healthcare.

In Maya’s transition “*From pained carer to empathic employer,”* the layers of vulnerability can be illustrated in her transition from family carer to employer when she reached a tipping point: she returned home after divorce to take up the responsibility to care for her mother who has dementia with no perceived suitable care available, which led her to employ a live-in carer. The challenges do not stop when the family carers become employers, however, because they are left alone and fully responsible to train the live-in carers (Liang, 2021). The limited support from the existing long-term care only exacerbates the exhaustion, conflicts and struggles experienced by the family employers as well as the care recipients (Basnyat & Chang, 2017; Chen, 2015). The participants’ narratives indicate that by transitioning into employers, they as family carers can recuperate from the compassion fatigue by incorporating the live-in carers as their burnout buffer. However, the live-in carer becomes a temporary yet permanent substitute: temporary because the live-in carer can only stay in the country for a limited time and permanent because to ensure continuous care in the household, the family employer will likely continue to hire live-in carers to take care of their older family member, one after another. The findings, therefore, provide a window of understanding into how the inadequate formal care services can exacerbate the vulnerabilities experienced by families with older family members that have extensive needs. Almost 80% of family carers in Taiwan have not utilized long-term care services in the past 12 months (Ministry of Health & Welfare, 2018, https://dep.mohw.gov.tw/dos/lp-5095-113-xCat-y106.html).

Maya reached the tipping point when she could not find what she perceived to be suitable care for her mother in the formal health care system. This led her to hire a live-in-carer, but even when she did this, she remained in a vulnerable situation. This is because her mother had severe dementia and needed care 24/7. Full caregiving responsibilities could not be given to the live-in carer because then the live-in carer would probably burn out and leave. In order to manage this vulnerable situation, Maya would have benefited from help from Taiwan’s formal services, but this did not happen. Structurally, family employers in Taiwan are punished for hiring a live-in carer from abroad (F. T. Y. Wang et al., 2006). They are excluded from a number of Taiwan’s formal services because they can afford to hire a live-in carer. The ideology behind this is the reaffirmation that filial responsibility should be maintained within the family, with minor formal healthcare services scarcely available for those whose families that do not function according to the ideal standard (L. J. Chen et al., 2015; F. T. Y. Wang et al., 2006).

### Achieving Person-Centred Care Within the Context of Multiple Vulnerabilities

The Indigenous participants in this study hired live-in carers so that their older family members could age at home and receive person-centered care despite their extensive care needs. Shahid et al. (2018) confirm that the key preference of the palliative care of Indigenous peoples in Australia, New Zealand, Canada and the United States includes dying close to or at home with the intimate involvement of family and integration of cultural practices. This is in line with Hoens and Smetcoren (2023) who highlight that the motivation for hiring a live-in carer is the need for aging in place, reducing the family carers’ workload and facilitating person-centered and continuous care toward the family member when they cannot provide this care themselves. The need for seeking person-centered care can be seen in Maya’s transition when she stressed that she did not want the physician to continue giving her mother sleeping pills, because that led to an unacceptable change in her mother’s behavior. It can also be seen in the same transition when Maya did not want her aunt to take care of her mother, because the aunt started to argue with her mother and did not understand the complexity of her mother’s disease. All this resulted in hiring a live-in-carer, because it was assumed that a live-in-carer could take the place of family members and offer person centeredness in their approach to care. The need for wanting to facilitate person centredness can also be seen in Yuma’s transition where she took her sister-in-law home from the nursing home because the personnel at the nursing home could not provide person-centered care nor address the sister-in-law’s loneliness due to being apart from her family. Person-centered care can be described as an approach that facilitates the care receivers’ right to self-determination and therefore a more respectful encounter between the receiver and the provider of the care services (McCormack et al., 2015). Family needs to provide person-centered care for their family member were in line with their values, and shaped by their culture and language. This study explicates the ways in which the Indigenous families negotiate cultural norms in arranging the best possible care for their family member.

In Kawas’s transition *“From exhausted carer to demanding employer”*, her mother’s increasingly challenging behavior and extensive care needs, extended beyond the family’s ability to provide the person-centered care that was desired, and therefore the decision was made to hire a live-in carer to provide this care as well as relieve some of their strain. This is also stressed by Horn et al. (2019) who assert that hiring a live-in carer contributes to a more person-centered care in addition to relieving the families of some of the care burden and preventing residential care. Moreover, it also facilitates continuity in care. The results in this study suggest that by becoming an employer, the family carers attempted to ensure that their older family members received continual and person-centered care in their own home.

### Seeking Culturally Safety While Navigating Colonial Relationships

The findings also indicate that cultural safety is multifaceted. In the cases presented the participants pointed out that pre-existing care options were “lacking empathy” (Maya) and producing “loneliness” (Yuma). The ramification may not be fully understood if this is portrayed as a personal preference problem. Indeed, a wealth of literature on cultural safety has evidenced that health and social service providers should be mindful of the characteristics that make people diverse and different, including the impact of colonial context (Baskin et al., 2020; Papps & Ramsden, 1996). In their search for the best possible healthcare for their family members, participants tried to establish culturally safe environments; this can also be seen in relation to what Williams (1999) calls an environment with shared respect, meaning, knowledge and experience through learning together and truly listening to each other. The transitions from family carer to employer indicate that cultural safety is influenced by layered colonization that hold potential for harm for both Indigenous peoples and migrant live-in carers. For example, Kawas considered installing cameras to monitor their live-in carer in her transition in order to try to ensure person-centered care. Moving from a caregiver to an employer, Kawas’ transition raises the question of cultural safety perceived by *whom*. Meanwhile, the others tried to create a safe milieu and protect the live-in carers from vulnerability. For example, Yuma invited the live-in carer to eat with the family although she was aware it was against the guidance. Yet, the fact that the families hires live-in carers may be indicative of how vulnerability is reproduced—even though they did not intentionally create additional vulnerabilities for live-in carers. That is, the findings point out a need for contextualizing what cultural safety mean when it comes to the employers and live-in carers embedded in the unsafe care system.

The Indigenous peoples of Taiwan have experienced trauma resulting from colonialism, namely, losing their ancestral languages, self-confidence and way of life (Teyra & Hsieh, 2022), which is precisely why they may be referred to as *vulnerable people*. They are victims of colonization, as they survived the trauma, tragedy and powerlessness that resulted from the Japanese and Chinese colonization (Barclay, 2018; Teyra & Hsieh, 2022). Cree Anishinaabe researcher Marcia Anderson contested that instead of *vulnerable people*, it is more accurate to say, “people we oppress through policy choices and discourses of racial inferiority” (Anderson, 2020). Indeed, in transitioning from family carers into employers, the pathways show the tendencies of reproducing vulnerability—an oppressed condition as a result of Taiwan’s care policies and discriminatory practices. This means that the participants in this study, in their attempt to try to find a solution to provide continued person-centered care for their family member with extensive care needs and for their family as a whole, may risk reproducing vulnerability.

## Conclusion

This study aimed to understand the transition from family carer to employer among Indigenous families in Taiwan who had older family members with extensive care needs. In the three different transitions, numerous dilemmas were revealed in navigating the care system. The revealed dilemmas were shaped by emotions, culture, finances, trust and communication. In addition, it should be recognized that families who feel morally obligated to follow the filial norm, and to fulfill this norm without having sufficient public-funded services is a form of exploitation.

The participants in this study struggled to provide person-centered care in line with their cultural values and beliefs, and when they are unable to provide this care themselves, within the family or in the care system, they turn to the live-in carer system. In the care system that is severely insufficient due to the unjust colonial distribution of services, the colonized risk becoming the colonizer and reproducing earlier vulnerabilities because they have the ability to hire a live-in-carer. This study contributes to illuminating the context-dependent concept of vulnerability, and more generally, the relationship between decoloniality and care.

The findings have some important implications. First, our study indicates that the transition from family carer to employer comes from a necessity, as family carers reached a breaking point when it is no longer possible to maintain responsibilities for care on their own. Employing a live-in carer helps families cope and retain the well-being of their family member, and offers a better chance of person-centered care for their older family member. Secondly, person-centered care in Indigenous families should be designed in a more wholistic way that ensures cultural safety. We cannot only think about the individual, but also the surrounding landscape and environment that enables the care (Ness & Munkejord, 2021). This awareness of contextualized person-centered care is highly relevant to nurses in varying health care facilities in and outside of Indigenous communities.
